# Impact of Stress on Epilepsy: Focus on Neuroinflammation—A Mini Review

**DOI:** 10.3390/ijms22084061

**Published:** 2021-04-14

**Authors:** Claudia Espinosa-Garcia, Helena Zeleke, Asheebo Rojas

**Affiliations:** 1 Department of Pharmacology and Chemical Biology, Emory University School of Medicine, Atlanta, GA 30322, USA; 2 Neuroscience and Behavioral Biology Program, Emory University College of Arts and Sciences, Atlanta, GA 30322, USA; helena.abebaw.zeleke@emory.edu

**Keywords:** epilepsy, stress, seizures, psychiatric comorbidities in epilepsy, depression, neuroinflammation, priming, microglia, NLRP3 inflammasome

## Abstract

Epilepsy, one of the most common neurological disorders worldwide, is characterized by recurrent seizures and subsequent brain damage. Despite strong evidence supporting a deleterious impact on seizure occurrence and outcome severity, stress is an overlooked component in people with epilepsy. With regard to stressor duration and timing, acute stress can be protective in epileptogenesis, while chronic stress often promotes seizure occurrence in epilepsy patients. Preclinical research suggests that chronic stress promotes neuroinflammation and leads to a depressive state. Depression is the most common psychiatric comorbidity in people with epilepsy, resulting in a poor quality of life. Here, we summarize studies investigating acute and chronic stress as a seizure trigger and an important factor that worsens epilepsy outcomes and psychiatric comorbidities. Mechanistic insight into the impact of stress on epilepsy may create a window of opportunity for future interventions targeting neuroinflammation-related disorders.

## 1. Introduction

According to the World Health Organization, epilepsy is a chronic neurological condition affecting around 50 million people worldwide, characterized by recurrent unprovoked seizures [1]. Among other factors, stress has been reported as the most frequent trigger for seizures in people with epilepsy [2,3,4,5]. Stress is defined as the physiological and behavioral response to an uncontrollable and/or unpredictable event [6]. Since humans differ in their response to stressful life events depending on duration, intensity, and type of stressor, accurate stress measurements in the clinic are complex [7]. Animal studies have shown that exposure to acute stressors in most cases, protect against seizures; while exposure to chronic stressors increases seizure risk and frequency contributing to enhanced anxiety behavior or a depressive state [8,9,10,11]. Depression is the most frequent psychiatric comorbidity with epilepsy, affecting up to 62% of people with epilepsy, but remains underrecognized and undertreated [12,13,14,15]. Very recent reports from the National Institute of Neurological Disorders and Stroke/American Epilepsy Society (NINDS/AES) Committee [16] and the International League Against Epilepsy (ILAE) Psychology Task Force [17] have emphasized the need for a better understanding of psychiatric comorbidities to improve epilepsy management and quality of patient life. 

Epilepsy and depression are associated with elevated brain inflammation, which can be induced by seizure activity as well as by behavioral, environmental, and physiological stressors [18]. The immune system plays a very prominent role in linking stress with depression [19,20,21,22,23]. Exposure to stress can sensitize or prime the inflammatory response to a subsequent insult, e.g., seizures, predisposing individuals to psychiatric comorbidities such as depression [24,25,26]. Numerous proinflammatory pathways are activated in depressed humans and stressed animals, including the NOD-like receptor pyrin domain containing 3 (NLRP3) inflammasome, which is constitutively expressed in immune cells such as microglia and other myeloid cells [18,20,23]. Although anti-inflammatory drugs have a huge potential to manage depressive symptoms associated with inflammation [27], a very recent multicenter, randomized clinical trial using two anti-inflammatory drugs (minocycline and celecoxib) failed to show a reduction in depressive symptom scores between placebo and drug-treated groups [28]. These results highlight the urgent need for using innovative study designs (e.g., match-mismatch designs) and specific outcomes to measure anti-inflammatory effects on the brain, as well as using more selective drugs that target both brain and peripheral inflammation in depressed patients, especially those diagnosed with epilepsy [29]. Here, we discuss preclinical and clinical evidence indicating stress as a crucial component to epilepsy disease progression. The need for proper care and treatment of people with epilepsy and comorbid depression is also emphasized with regard to stress management. Preclinical and clinical studies on the effects of stress in epilepsy with comorbid depression were identified from electronic searches using the PubMed database. Briefly, our search was restricted to studies addressing either physical or social stressors; we recognized that psychological stress is difficult to model in animals and therefore was excluded from our discussion. We screened to identify additional reports including the effects of stress and seizures on microglia and neuroinflammation.

## 2. Acute Stress in Epilepsy

Acute stress is defined here as the biological and psychological sequelae associated with any threatening stimulus presented for a short and distinct period. In this review, we consider any acute stressor that is presented at a certain interval and on multiple occasions to be an acute repetitive stressor due to it subsiding for periods between subsequent exposures, unlike a chronic stressor which is continuously present. For simplicity, we only discuss acute stimuli that are associated with either a psychogenic (psychological strain/tension) or neurogenic (physical pain/tension) stress effects, excluding stimuli that would not induce the biological stress response in non-epileptic animals and humans (unconditioned visual cues, smells, sounds, etc.). In non-human animals, acute stress may be induced by restraint, foot-shock, or forced swimming (Table S1). Similarly, acute stress can be brought on by a variety of experiences in people with epilepsy, including sudden loss of a loved one, accidents, or less emotionally salient experiences like arguments and difficult interviews (Table S2). In this section, we discuss the effects of both acute stress and acute repetitive stress on epileptogenesis, the process by which a normal brain becomes chronically prone to seizures, and seizure susceptibility in people with epilepsy and in animal models of epilepsy. 

### 2.1. Acute Stress Effects on Epilepsy in Humans

Previous studies have indicated that stress is one of the most common self-reported seizure precipitants [3,4]. However, many of these self-report studies fail to discriminate between acute and chronic stressors, which complicates linking findings from the experimental context to the experiences of epileptic patients. An exception is a study published in 2014, in which participants were asked to list whether their stress-precipitated seizures were due to acute (minutes to hours in duration) or chronic (days to months in duration) stressors [30]. The authors found that 219 out of the 266 epileptic participants endorsed stress as a seizure precipitant, and 68% of these 219 patients specifically endorsed acute stress as a contributor.

Furthermore, it is difficult to validate results from human studies because investigators often have participants rank their stress levels using generic categories, making it difficult to determine whether participants within a certain group are actually experiencing similar intensities and frequencies of stressful situations. In an attempt to evade this problem, several studies exploring the effects of stress on epilepsy have examined the effects of widespread stressors that have a common impact on a certain population (i.e., natural disasters), but even these findings are complicated by the differences in individual appraisal of such emotional stressors. Additionally, traumatic events like natural disasters and war experiences often have long-lasting after-effects (i.e., homelessness) that make it nearly impossible to describe them as exclusively acute.

An alternate approach to studying the effects of acute stress on human epilepsy is to present epilepsy patients with controlled psychological stressors or empathetically stressful stimuli and record the resulting brain activity and behavioral manifestations (Table S2). This method was employed by investigators in 1959 to reveal abnormal EEG activity following a stressful interview in a small study of epileptic patients (N = 39) [31]. Another early study found that video and audio recordings of stressful social interactions were capable of producing seizures in 100% of the five epileptic participants monitored [32]. Studies like these have waned over the years due to ethical concerns of precipitating seizures in patients, but they provide useful insight into the effects of controlled stressors. Interestingly, emerging evidence in the form of individual case reports and preclinical studies suggest that high-intensity, demanding physical exercise reduces seizure activity [33,34]. Though it is possible that the stress component of physical exercise is responsible for this phenomenon, activation of the stress response is only one of several relevant neurophysiological processes (i.e., release of neurotrophic factors, up-regulation of GABAergic systems) promoted by physical exercise [35]. More work needs to be done to discern whether the acute stress associated with physically demanding exercise is important for reducing seizure activity.

### 2.2. Acute Stress in Animal Models of Epilepsy

#### 2.2.1. Acute Stress in Epileptogenesis

Although this review focuses primarily on the epilepsy-modifying effects of acute and chronic stressors, there is substantial literature detailing the effects of acute stress on the antecedent processes of initial seizure induction and epileptogenesis. In these experiments, an acute stressor is presented to the animal prior to primary chemical or electrical seizure induction. Investigators use measures such as latency to the first seizure, seizure threshold, functionality of various brain circuits, and relative levels of neuromodulatory compounds associated with the first seizure or status epilepticus (SE) onset to better understand the anti- or pro- convulsive priming effects of stress. These studies have yielded widely varying results with findings that describe both anti-convulsive and pro-convulsive stress effects. The discrepancy between results can be attributed to the variance associated with the use of different acute stressors.

Evidence for a pro-convulsive effect of acute stress has more recently been described in experiments with zebrafish, which have revealed that exposure to conspecific alarm substance, an intense acute stressor, prior to initial pentylenetetrazol (PTZ)-induced SE potentiates seizure susceptibility and the intensity of convulsive behaviors [36]. However, acute swim stress prior to seizure induction by five different chemoconvulsants has been shown to increase seizure and mortality thresholds in mice, an anti-convulsive effect that is consistent with other studies of acute swim stress [8,37]. Researchers have since utilized other stress paradigms in mice, including acute restraint stress and acute foot-shock stress, to support the assertion that acute stress prior to the first seizure has an anti-convulsive effect [38,39]. This anti-convulsive effect of acute stress is suspected to involve activation of mineralocorticoid receptors, which promotes long-term potentiation in the hippocampus of mice after seizure activity [40]. Altogether, the existing literature more strongly suggests an anti-convulsive effect of acute stress on primary seizure induction and epileptogenesis. Furthermore, the differences between the findings of various groups of investigators demonstrates the potentially confounding effects of different stress paradigms, species studied, and methods of seizure induction on the comparability of these results [8].

#### 2.2.2. Acute Repetitive Stress in Epileptogenesis

Several studies have also investigated the effects of acute repetitive stress, as opposed to a single presentation of an acute stressor, on primary seizure induction and epileptogenesis. In an investigation of acute stress on the response to GABA-related and unrelated convulsants in mice, investigators discovered that repeated exposure to swim stress for five consecutive days before seizure induction conferred a smaller anticonvulsant effect against picrotoxin-induced SE than did the single exposure to acute swim stress [37]. Repetitive swim stress has also been found to alter epileptogenesis by decreasing the number of benzodiazepine receptors in various limbic brain regions and decreasing the anticonvulsant potency of the benzodiazepine clonazepam in mice [41]. Acute repetitive social defeat stress prior to kainic acid-induced SE in rats also results in a reduction of seizure threshold and facilitation of epileptogenesis that is dependent on brain-derived neurotrophic factor (BDNF) levels [24,42]. Collectively, these findings demonstrate the importance of consistency across methods used in experiments exploring the modifying effects of acute stress on epileptogenesis.

#### 2.2.3. Acute Stress in Chronic Phase of Epilepsy

**Figure 1:**
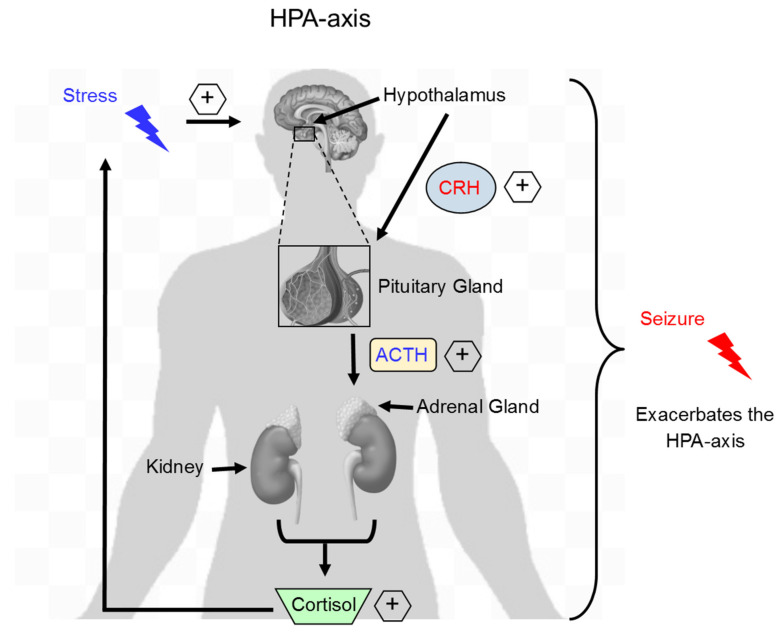
Stress activates the HPA-axis and exacerbates seizure occurrence. Exposure to stress results in hypothalamic release of CRH, which binds its receptors located on the pituitary gland and then subsequent release of ACTH. ACTH binds to receptors located on the adrenal gland and stimulates adrenal release of the stress hormone cortisol. During acute stress, cortisol exerts a negative feedback to CRH and ACTH release. In contrast, during chronic stress, increased cortisol levels persist leading to sustained HPA-axis activation. During a subsequent insult, such as a seizure, activation of the HPA-axis is exacerbated and contributes to future seizure susceptibility and worsened outcomes. HPA, hypothalamus-pituitary-adrenal axis; CRH, corticotropin-releasing hormone; ACTH, adrenocorticotropic hormone.

The anticonvulsive properties of stress in the context of epileptogenesis sharply contrasts its almost completely deleterious effects in the chronic phase of epilepsy (Table S1). Studies in the 1980s investigating the interaction between acute stress and epilepsy found that immobilization and handling stressors exacerbates convulsive behavior and epileptiform spiking in some, but not all rats treated with opiate-based convulsants [43,44]. Correspondingly, acute immobilization stress significantly increases interictal discharges in hippocampal kindled rats [45]. However, experiments with amygdala-kindled rats demonstrated that acute social defeat stress decreases the severity and duration of motor seizures [46]. More recent studies have used multiple models of genetic epilepsy to correlate acute stress and seizure susceptibility [8]. In experiments utilizing a rat model of genetic absence epilepsy, epileptic rodents exhibited a more than three-fold increase in spike-wave discharges (SWD) in response to exogenous corticosterone, which is an important mediator of the stress response (see Figure 1) [47]. Similarly, mice with genetic focal epilepsy (EL mice) have also demonstrated heightened epileptiform EEG activity in response to tail suspension stress [48]. An additional model of genetic absence epilepsy in mice (Scn8a voltage-gated sodium channel mutation) was used to describe a similar effect, in which 20-minute acute restraint stress increased the recurrence of spontaneous SWDs [49]. Taken together, animal studies show that subjecting rodents to acute stress in the chronic phase of epilepsy promotes seizure activity.

#### 2.2.4. Acute Repetitive Stress in Chronic Phase of Epilepsy

Although some earlier studies employing handling stress did not specify the frequency of acute stressor presentation, repeated presentation of acute stressors has been shown to significantly modulate the seizure precipitating effects of acute stress in epileptic rodents (Table S1) [3,44]. A cumulative pro-convulsive effect of daily foot shock on SWD intensity was demonstrated in WAG/Rij rats wherein exacerbations of SWDs were revealed only after three consecutive days of foot shock [50]. EL mice have also demonstrated unique increases in plasma corticosterone after repeated exposure to tail suspension and foot shock stressors, with values that exceed those of control mice subjected to the same stressors [48]. Thus, the evidence shows that repeated exposure exacerbates the proconvulsive effects of acute stress as well as its ability to further dysregulate the stress response in epileptic rodents.

### 2.3. Acute Stress and Hormonal Changes

Researchers investigating stress-related effects on epilepsy have also conducted studies to determine the hormonal basis for the negative effects of acute stress. The literature consists of conflicting findings, especially regarding the ability of acute stress to produce hyperactivity of the hypothalamus-pituitary-adrenal (HPA) axis in rodent models of epilepsy (Figure 1). For example, hippocampal kindling exacerbates cortisol and adrenocorticotropic hormone (ACTH) release in Sprague-Dawley rats, but diminishes basal levels of cortisol in Wistar rats, in models of acute stress [8,51,52]. Animals with epileptic predispositions, like the Mongolian gerbil and the epilepsy (EL) mouse, have also demonstrated upregulation of HPA-axis mediators, including corticotropin-releasing factor (CRF) and cortisol, in response to acute stressors [8,48]. The existing evidence suggests that differences between findings may be the result of variation in methodology, and that mediators of the HPA axis are involved in the propagation of seizure activity [53].

Altogether, studies implementing acute stress paradigms in combination with animal models of epilepsy have revealed several interesting characteristics regarding the relationship between stress and seizures. The evidence describes acute stress prior to epilepsy-inducing brain injury as well as during the epileptogenesis phase as anti-convulsive. On the other hand, acute stress after epilepsy onset is consistently cited as having a deleterious effect on seizure progression. This effect is particularly relevant in the clinic, as patients with epilepsy are at greater risk of encountering acute stressors like car accidents and falls due to their condition compared to people without epilepsy [54,55].

### 2.4. Neuroinflammation and Acute Stress in Epilepsy

The neuroinflammation associated with exposure to acute stress is important to examine as a potential mechanism by which stress results in greater seizure susceptibility. In this review, neuroinflammation refers to the transient but robust inflammatory response in the central nervous system (CNS) characterized by a change in expression of inflammatory mediators including cytokines, chemokines, cell adhesion molecules, prostaglandins, prostaglandin-ethanolamides, pro-inflammatory enzymes as well as gliosis, all as a result of enhanced seizure activity. The change in the levels of these mediators persists for days to weeks following the precipitating event. The robust inflammatory mediator burst in the brain following status epilepticus is presumed to cause secondary damage in the brain and increase the likelihood of repetitive seizures contributing to epileptogenesis. Therefore, reducing neuroinflammation via anti-inflammatory therapy following status epilepticus may prevent the development of spontaneous recurrent seizures or alter the severity of epilepsy.

**Figure 2:**
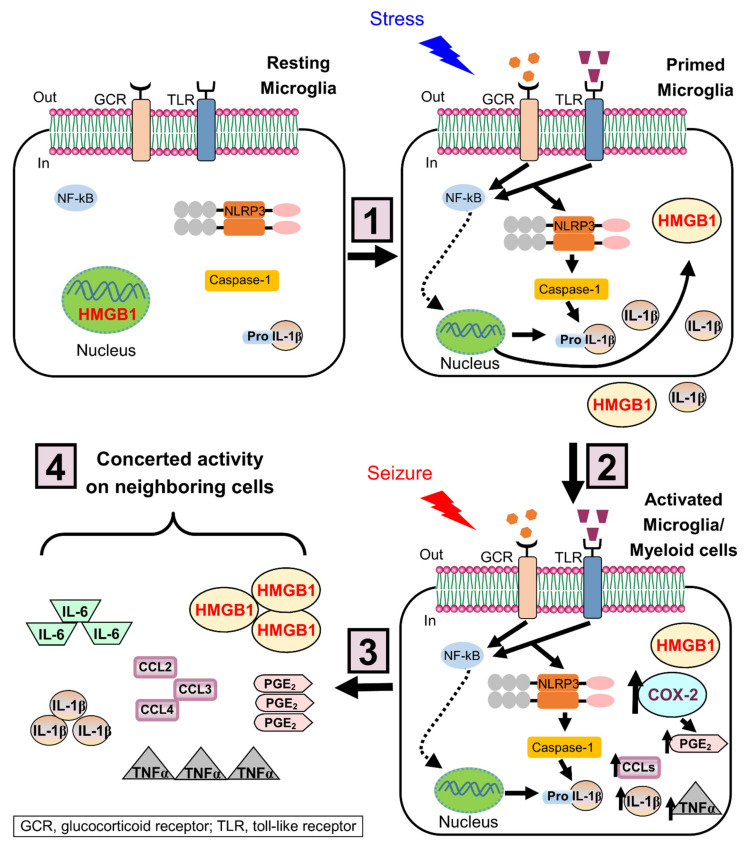
Priming effects of stress on NLRP3 inflammasome in microglia. The mechanism of stress-induced microglial priming involves a 2-hit process: (**1**) In the first hit, stress increases the levels of glucocorticoids, and induces the alarmin HMGB1 to be translocated from the nucleus to the cytoplasm and then released to the extracellular space. Once released, HMGB1 binds its receptors (e.g., toll-like receptors, TLR) and induces upregulation of the NOD-like receptor NLRP3 expression in primed microglia, likely through the TLR/NFκB signaling pathway. (**2**) In the epileptic brain, seizures (second hit) activate primed microglia. The inflammasome complex is assembled and caspase-1 cleaves pro-IL-1β, resulting in secretion of mature IL-1β. (**3**) Besides NLRP3 inflammasome activation, seizures increase key inflammatory mediators, such as COX-2, PGE_2_, chemokines (CCLs), TNFα, and IL-6. (**4**) These inflammatory mediators act in concert with the subsequent activation of neighboring cells and the recruitment of myeloid cells, leading to a potentiated inflammatory response that worsens the brain injury. HMGB-1, high mobility group box-1; NLRP3, nucleotide-binding domain and leucine-rich repeat containing family, pyrin domain containing 3; TLR, Toll-like receptor; NF-κB, nuclear factor of kappa light polypeptide gene enhancer in B-cells; COX-2, cyclooxygenase 2; PGE_2_, Prostaglandin E2; CCLs, C-C motif chemokine ligands; TNFα, tumor necrosis factor alpha.

Repetitive acute stress exposure is a precipitant of microglial activation and peripheral monocyte recruitment to the CNS; however, fewer studies have explored the neuroinflammatory effects of a single, acute stressor [56,57,58]. HPA-axis activation, a rough biological correlate of the acute stress response, has been shown to disrupt the blood-brain barrier, thus further facilitating the migration of monocytes to the brain [59]. Recently, it was demonstrated that an increase in expression of pro-inflammatory mediators, including interleukin 16 (IL-16) and nuclear factor kappa light chain enhancer of activated B cells (NF-kB), is also observed in mice subjected to a single exposure of 12 h to cold stress, which is a relatively acute, non-repetitive stress paradigm [60]. The release of these proinflammatory molecules in the brain after exposure to an acute stressor has been linked to activation of the NLRP3 inflammasome (Figure 2) [21,61]. NLRP3 inflammasome activation has also been observed in rats subjected to acute repetitive social defeat stress [62]. Collectively, these studies support the notion that stressors induce a neuroinflammatory response in the rodent brain. Although the large majority of these studies are not carried out in epileptic rodents, it is likely that the stress-induced neuroinflammation exacerbates the established proinflammatory effects of seizure activity to produce a hyper-inflammatory microenvironment. This hyper-inflammatory state may explain the increased risk that epilepsy patients have for developing inflammation-related psychiatric conditions, including depression (discussed below). The uniquely damaging neuroinflammatory microenvironment produced by the combination of stress and seizure activity highlights the need for anti-inflammatory treatments in the management of epilepsy.

## 3. Chronic Stress in Epilepsy

Chronic stress is another type of generally classified stress. If left untreated, chronic stress may have the greatest impact on an individual’s health. Chronic stress is defined here by us as the prolonged and constant (months to years for humans) feeling of emotional or physical tension caused often by a traumatic experience or heightened emotional state. Chronic stress occurs when the intensity and frequency of a stressor is so high that the body does not have an adequate chance to activate a physiological relax response, leaving an individual in a constant state of physiological arousal. This constantly heightened state affects the entire body either directly or indirectly and can be detrimental in pathophysiological conditions such as epilepsy. Although the individual response to chronic stress varies, if the stress persists, an individual may enter a depressive state. In humans, chronic stress exists in two general forms based on the nature of the stressor. In humans, one form of chronic stress has physical origins and the other form of chronic stress has psychological origins. For example, chronic stress in humans can be caused by everyday life experiences related to emotions, environmental stressors, relationship stress, and work-related stress. In this section, we discuss the impact chronic stress has on neuroinflammation and epilepsy prior to depression. Although many patients suffering from epilepsy believe that a stressful event or multiple stressors may have caused the initial development of epilepsy and there is a wealth of studies investigating the role of stress in epileptogenesis, here we focus on the role of chronic stress as a seizure-precipitating event in clinically diagnosed people with epilepsy.

### 3.1. Chronic Stress and Seizure Occurrence in Humans

People with epilepsy often report that everyday stressful events are the most common triggers of seizures [3,63,64,65,66]. A vast majority of the clinical studies investigating whether stress causes seizures are retrospective and confounded by patient self-reporting of seizures and the various types of stressful events leading to intrinsic bias. Although there are prospective studies investigating the effect of chronic stress on seizure occurrence, we believe that the results of these studies can be misleading due to difficulty determining whether the increase in seizures is due to the stressor or another confound that could also be stressful yet difficult to measure, such as sleep deprivation, alcohol use, poor diet, mismanaged medication, or mood disorders (i.e., increased anxiety). Nevertheless, these clinical studies have provided valuable information regarding the relationship of stress, psychological health, and epilepsy. There is only a small subset of clinical studies investigating the effects of chronic stress on seizure susceptibility with the consistent finding that chronic stress is proconvulsive.

Chronic stress caused by war, a terrorist attack, or natural disasters increase seizure frequency in adult patients [67,68,69]. These were relatively small clinical studies with 66–117 patients. More recently, a longitudinal study of 558 patients demonstrated a relationship between chronic stress, depression, anxiety, as well as seizure recency (time passed from the last seizure) and seizure frequency [70]. The authors of the study concluded that stress, depression, and anxiety state separately predicted a change in patient seizure recency and frequency; however, depression mediated the relationship of anxiety, stress and seizure frequency. In a separate study published five years later, investigators reported that among epilepsy patients (266 total patients) who were stressed, 85% endorsed chronic stress as a seizure precipitant and the stressed patients believed that they could occasionally predict seizure onset [30]. There appears to be a strong positive correlation between chronically elevated stress levels and increase seizure frequency; however, these studies are confounded by other mood disorders. For example, patients that experience chronic stress often experience high anxiety and depression that could facilitate seizures. Depression and anxiety are common comorbidities of epilepsy and there are complex relationships between these disorders that are still being investigated. Another confounding factor related to whether chronic stress lowers the seizure threshold is the theory that repetitive acute stress often becomes chronic stress [71,72]. The theory proposes the existence of a brain circuit of stress involving the hippocampus, amygdala, dorsal raphe nucleus, and the entorhinal cortex. Through the network circuitry, activation of these brain regions and a heightened state of excitability causes repetitive acute stress to transition to chronic stress. The loss of neurons in the dentate granule cell layer of the hippocampus is partially responsible for memory loss and is key to the pathophysiology of temporal lobe epilepsy and the brain circuit of stress [72]. Therefore, loss of dentate granule cells caused by seizures leads to an impaired brain circuit of stress that in a feedback mechanism can result in more seizures.

### 3.2. Chronic Stress in Animal Models of Epilepsy

The majority of animal studies investigating the effects of chronic stress on seizure occurrence involve rodents whose psychological response to a chronic stressor is difficult to ascertain. Therefore, investigating the effect of chronic stress on seizure occurrence in rodents often involves the use of prolonged physical stressors to induce chronic stress rather than psychological stressors. The two most common physical stressors used to induce chronic stress in animals are social isolation and restraint or immobilization. Social isolation consists of housing rodents individually in a cage. Similar to humans, rodents are social beings and social isolation causes loneliness that can be very stressful when prolonged. Although social isolation is classified as a physical stressor, there is a component of psychological stress as well. Social isolation stress causes behavioral and endocrine responses in adult rodents leading to anxiety, aggression, and nervousness [73]. Recently, it was demonstrated that social isolation of rats and mice results in a higher level of stress and an increase in seizure frequency compared to animals maintaining social contact [74]. In this study continuous (24/7) electroencephalography (EEG) recordings beginning six weeks after status epilepticus (SE) revealed that rats developed epilepsy following pilocarpine-induced SE and socially isolated rats displayed seizures more frequent (16 times) compared to rats socially housed [74]. Chronic social isolation lowers the seizure threshold, making it more likely for seizures to initiate [75,76]. During chronic social isolation, the brain concentration of neurosteroids such as allopregnanolone, pregnenolone, and progesterone decreases [77]. A reduction in brain neurosteroids can lead to impaired neuronal inhibition, as neurosteroids are potent positive modulators of GABA_A_ receptors. The altered GABA receptor modulation is a proposed mechanism by which chronic stress may reduce the seizure threshold, increasing the frequency of seizures in epilepsy patients.

Chronic restraint is also used in rodents as a method of chronic stress involving prolonged movement restriction, resulting in a lack of physical activity and a depressive state. Chronic restraint induces a myriad of functional and structural changes in the brain of rodents [78]. For example, studies have shown that chronic stress induced by prolonged physical restraint causes a long-lasting increase in corticosterone levels and reduces the seizure threshold [79,80,81,82,83]. The increased seizure susceptibility observed in these multiple rodent convulsant models is attributed to the elevated circulating corticosterone levels resulting from chronic restraint stress. If the restraint stress persists, the animal may enter a depressive state. Nevertheless, the occurrence of repeated seizures appears to be altered by the degree of chronic stress experienced by the animal. Despite studies demonstrating experimental evidence of a lowered seizure threshold following chronic stress, the precise mechanisms by which chronic stress contributes to the precipitation of seizures is still unclear and needs further investigation.

Unlike social isolation and physical restraint, environmental enrichment has been shown to reduce stress levels in rodents [84]. Environmental enrichment promotes social interaction and sensory stimulation by enhancing the social or physical surroundings of a human or animal by placing objects such as toys, structures, or other social beings in the same environment. The reduced stress levels in rodents as a result of environmental enrichment has been shown to protect against seizures [85,86,87,88,89,90]. Environmental enrichment also ameliorates depressive behavior in the forced swim test after seizures in male rats [91]. Environmental enrichment enhances molecular and morphological changes in the rodent brain that play an important role in epilepsy. In addition to the reduced frequency of seizures, additional benefits of environmental enrichment in rodents include reduced corticotropin-releasing factor expression, improved brain function, enhanced neural plasticity in brain areas regulating emotion, and cognitive enhancement. Rodents housed in an enriched environment tend to display less neuronal damage, anxiolytic behavior, and enhanced cognition, compared to rodents placed in an environment lacking enrichment [85,86,87,88,89,90]. The lowered seizure susceptibility displayed by rodents in an enriched environment is attributed to the combination of the aforementioned effects as well as a lowered stress level. The relationship between environmental enhancement and epilepsy was recently discussed in detail in a review article [92].

### 3.3. Neuroinflammation and Chronic Stress in Epilepsy

A major goal in epilepsy research is to determine the key events that occur in the brain after an injury, such as stroke, trauma, prolonged febrile seizures or status epilepticus that predispose it to develop spontaneous recurrent seizures. Rational therapeutic intervention for epilepsy requires an understanding of the mechanisms responsible for epileptogenesis. Despite successes in developing therapies for existing epilepsies, our understanding of the sequence of events, such as those after a brain injury that causes a normal brain to become epileptic is lacking. There are very limited clinical cases reporting the pathology of brain tissue with regard to inflammatory mediators in humans; however, serum levels of inflammatory mediators such as interleukin-1 beta (IL-1β) and high mobility group box 1 (HMGB1) have been shown to correlate with seizure severity in children with febrile seizures (41 total patients) (Figure 2) [93]. Additional evidence of a potential role of inflammatory mediators in epilepsy is derived from clinical studies wherein inflammatory mediators were measured in the cerebrospinal fluid of 14 epileptic patients with severe seizures and compared to 14 patients with other neurological diseases [94]. Although human studies investigating the relationship between neuroinflammation and seizures are ongoing, the vast majority of information on this subject comes from numerous animal studies.

Prolonged status epilepticus initiates molecular and cellular events in the brain that eventually culminate in the appearance of spontaneous recurrent seizures. These events include but are not limited to selective neuronal degeneration, inflammatory signaling and gliosis (neuroinflammation), axonal sprouting, neurogenesis, and new synapse formation resulting in enhanced synaptic efficacy [95]. Some of the most difficult to treat forms of human epilepsy are associated with severe neuroinflammation (especially in children), for example epilepsy caused by encephalitis or brain abscess. Inflammation is also a feature of long-standing epilepsy in sclerotic human hippocampus [96,97] in studies involving small sample sizes of 6 and 18 epileptics patients. Although changes in the expression of numerous inflammatory mediators occur after seizures, the role of post-seizure inflammation in the development of epilepsy is ambiguous and has received little attention until recently. For example, a PubMed query to identify review articles on the topic of “epilepsy and neuroinflammation” yielded 232 results from 2003 to 2021, most of which were published in the last decade (82% from 2012 to current).

In the epileptic brain, each seizure results in a neuroinflammatory response that is believed to contribute to lowering of the seizure threshold, resulting in the progression and worsening of epilepsy [98,99]. Therefore, targeting neuroinflammation may also have an epilepsy modifying effect. Currently, there is a relatively short list of key molecular inflammatory mediators as druggable targets to reduce neuroinflammation after seizures (see additional manuscripts [100,101] for a more detailed review of these targets). The propagation of pro-inflammatory mediators following seizures promotes hyperexcitability by acting directly on the receptors expressed on the surface of neurons or indirectly via interactions between neurons and other cell types such as microglia, monocytes, and astrocytes. For example, regulation of the interleukin-1 receptor pathway has been shown to alter seizures in experimental animal models [102,103,104,105,106,107], resulting in the candidacy of the IL-1 receptor as a therapeutic target for epilepsy.

A key feature of neuroinflammation is gliosis or the activation of glial cells. Microglia and astrocytes are activated by seizures displaying a change in morphology and metabolic reactions [108,109]. Astrogliosis (astrocyte activation) and microgliosis (microglia activation) is often revealed by more intense immunohistochemical staining of glial fibrillary acidic protein (GFAP) and ionized calcium-binding adapter molecule 1 (Iba1), respectively, after seizures. The morphological change of glial cells after seizures is also accompanied by an increase in mRNA for GFAP and Iba1. Activation of glial cells serve to buffer the extracellular space around neurons regulate potassium ions, and release inflammatory mediators to mitigate the ensuing injury. During SE microglia, astrocytes, endothelial cells and infiltrating peripheral immune cells produce and release cytokines such as IL-1β, interleukin-6 (IL-6) and TNFα as well as chemokines like chemokine C-C motif ligands 2 and 3 (CCL2 and CCL3) and numerous other protective factors (Figure 2) [110]. These inflammatory mediators then act on nearby receptors expressed on neurons, astrocytes, and microglia activating a multitude of cellular signaling pathways. Microglia have two activation states: the classical M1 phenotype and the alternative M2 phenotype. M1 microglia contribute to the generation of a neurotoxic environment by expressing pro-inflammatory mediators. In contrast, M2 microglia promote tissue protection and repair by expressing anti-inflammatory mediators and neurotrophic factors [111]. In rodents, microglia co-express M1 and M2 markers following kainate- and pilocarpine-induced SE. These findings highlight the complexity of M1/M2 microglial activation in the epileptic brain [112]. In the epileptic brain, the microglial inflammatory response has a dual role. In the acute phase, examined at 3 days after SE, the microglial response is protective, helping to clear cellular debris by phagocytosis at the injury site, but this protection is short-lasting. In the chronic phase, examined at 5-12 months after SE, persistent microglial activation is detrimental for recovery and is associated with neurotoxicity and recurrent seizures [111,112,113]. Further studies are needed to identify the effects of stress on microglial M1/M2 activation states following seizures.

Seizures also result in breakdown of the blood-brain barrier (BBB) that lines the microvasculature of the CNS. The BBB plays a critical role in homeostasis in the brain and is comprised of endothelial cells, astrocytes, pericytes, and microglia [114,115]. The loss of integrity of the BBB is a significant event following seizures, as this allows for the passage of peripheral macromolecules and cells into the CNS. In animal models of SE and epilepsy breakdown of the BBB along with recruitment through chemokine signaling leads to infiltration of peripheral immune cells such as neutrophils and monocytes into the CNS [116,117]. The function of these cells upon entry into the CNS is not fully understood. However, there is evidence demonstrating that the presence of these cells results in deleterious consequences. For example, it has been shown that blood-derived monocytes promote brain inflammation and exacerbate neurodegeneration after SE induced by pilocarpine in mice [116]. Similarly, activated neutrophils once in the CNS following trauma promote further degradation of the BBB by physically altering cell membranes and releasing enzymes to alter the microvasculature [118,119]. Furthermore, patients with epilepsy have been found to have higher numbers of activated neutrophils in the CNS compared to non-epileptic patients [117] and this is potentially due to the high pro-inflammatory environment driven by the release of pro-inflammatory mediators by microglia and astrocytes during SE. The presence of these peripheral neutrophils also increases oxidative damage in the injured brain. Taken together, these changes associated with the loss of integrity of the BBB promotes epileptogenesis and facilitates the reoccurrence of seizures.

Despite numerous studies involving animal models of SE and epilepsy, little success has been achieved clinically by targeting neuroinflammation to combat epilepsy. However, there is epidemiological evidence demonstrating that long-term administration of non-steroidal anti-inflammatory drugs (NSAIDs) could reduce the risk of developing epilepsy [120]. Clinical evidence also exists demonstrating the use of NSAIDs to control or modify seizures in patients suffering from Sturge-Weber syndrome (SWS), which is a congenital neurological disorder that often involves malformation of blood vessels in the pia mater and underlying cortex [121]. There are also active projects investigating the benefit from pharmacological interventions targeting the inflammatory response caused by seizures in clinical and experimental epilepsy. For example, the IL-1R1 receptor antagonist Anakinra was shown to reduce seizure susceptibility in a mouse model of traumatic brain injury (TBI) [122] and reduce seizure frequency and memory deficits in a mouse model of autoimmune encephalitis [123]. Similarly, targeting of the TNFα receptor with the monoclonal anti-TNFα antibody Adalimumab reduced seizures in epileptic patients [124]. Inhibition of the EP2 receptor downstream in the COX-2 signaling pathway has been shown to reduce neuroinflammation and gliosis, resulting in a beneficial outcome in rodents following SE [125,126,127,128]. The early evidence suggesting a benefit of these therapies to alter epilepsy progression is encouraging and promote further investigation into anti-inflammatory therapies to combat epilepsy progression.

The effects of chronic stress on persistent inflammation in the periphery is well documented to exacerbate a barrage of health problems including diabetes, rheumatoid arthritis, and cardiovascular disease. However, much less is known about the relationship between chronic stress and neuroinflammation in epilepsy, which is now being heavily investigated, especially for the benefits of anti-inflammatory therapies. Together, chronic stress and neuroinflammation have a synergistic relationship in epilepsy. In the brain, chronic stress leads to an increase in stress-related hormones such as cortisol and corticotropin releasing factor (Figure 1) that can affect neuroinflammation. In summary, chronic stress has a direct effect on brain cells as it has been shown to change microglial morphology and enhance microglial function in seizure sensitive limbic regions of the brain such as the hippocampus, prefrontal cortex, and amygdala [108,109]. However, chronic stress also causes leakiness of the BBB [129,130], which could result in a number of consequences, such as infiltration of peripheral myeloid cells like monocytes and macrophages, and release of proinflammatory cytokines (IL-1β, IL-6 and TNFα) and chemokines [CCL2, 3, 4, C-X-C motif chemokine ligand 10, (CXCL10)] to aid in the recruitment of peripheral cells into the brain similar to that observed following SE. Therefore, chronic stress exacerbates damage and promotes the occurrence of seizures in the epileptic brain likely via a neuroinflammatory response.

## 4. Epilepsy and Depression

Depression is the most frequent psychiatric comorbidity in epilepsy. The Neurological Disorders Depression Inventory for Epilepsy (NDDI-E), a six-item questionnaire, has been validated to screen for depression in people with epilepsy [131,132]. In fact, the risk of developing depressive symptoms is increased almost 2-fold in people with epilepsy compared to healthy controls, and the disability rate and mortality are higher in epilepsy patients with depression [12,13,14,15,133]. Therefore, more effective diagnosis and treatment for people with epilepsy and comorbid depression are urgent unmet needs. However, the treatment of depressive symptoms in people with epilepsy is challenging. While some antiepileptic drugs can lead to depressive episodes through different mechanisms, including potentiation of GABA neurotransmission, folate deficiency, or pharmacodynamic interactions [134], some antidepressants can increase the risk of seizures [135]. Extensive review of results from 10 clinical trials [14], suggests that psychotherapy interventions should be considered as an alternative in patients with pharmacoresistant epilepsy, but a more in-depth investigation is needed to confirm these findings.

### 4.1. Animal Studies of Epilepsy and Comorbid Depression

In rodents, the forced swim test (FST) and the sucrose preference test (SPT) are traditional behavioral tests used to measure stress-induced depressive-like behaviors [136]. In the FST, animals are placed in a cylinder filled with water, from which they cannot escape. The experimenter records the immobility, which is characterized by floating in the water with only movements necessary to keep the nose above the surface. Immobility in the FST originally indicated “as a state of despair” is considered depressive-like behavior [137]. However, a recent notice from the National Institutes of Mental Health (NIMH) recommends the use of models “for” addressing neurobiological questions rather than models “of” specific mental illnesses [138]. Recently, a group of investigators suggested that immobility in the FST should be interpreted as a coping strategy for inescapable stressors, rather than depressive-like behavior [139]. On the other hand, the SPT is based on the animal’s natural preference for sweets, and a low sucrose consumption reflects anhedonia or lack of pleasure [140]. Exposure to chronic stress generally leads to longer immobility times in the FST and lower sucrose consumption in the SPT [141], but discrepancies in outcomes can be attributed to differences in nature of stress, severity of stress, exposure parameters [142], and resilience [143]. For example, after chronic exposure to social defeat, some rats exhibited submissive behaviors, indicating vulnerability to stress; while others exhibited resistance to submission, indicating that some animals developed resilience or coping behaviors [144]. Resilience in humans has been also shown to occur in the context of chronic stress [145]. These studies indicate that future challenges exist for conceiving new paradigms to dissect the complex connections between chronic stress and depression [146].

Numerous studies in rodents have demonstrated that status epilepticus (SE) leads to depressive-like behavior [147]. For instance, male rats subjected to LiCl and pilocarpine exhibited an increased immobility in the FST and a reduced saccharin preference [148]. In this study, the common antidepressant fluoxetine was not effective at improving depressive-like behaviors in SE animals, indicating pharmacological resistance. Similarly, pharmacoresistant anhedonia-like behavior was observed in SE mice subjected to pilocarpine or kainate [149]. Further studies [89,150,151,152] support the development of comorbid depressive-like behaviors in the chronic phase of epilepsy. Moreover, additional studies confirmed that seizures induced the activation of the HPA axis and elevated levels of the stress hormone corticosterone correlated with the severity of depressive-like behaviors in rodents [153,154]. A recent report indicated that epileptic mice showed anxiety-like and hyperactive behaviors, as well as increased anhedonic behaviors, but no difference in immobility time [155]. The authors suggested that stress circuits are disrupted in this epilepsy model. Disparities in cognitive and behavioral comorbidities in epilepsy may be due to differences in the severity, convulsant used, rat/mouse strain used, age, and induction protocol [156].

### 4.2. Sex Differences in Comorbid Depression among People with Epilepsy

There are well-established sex differences in psychiatric comorbidities associated with epilepsy [157]. For example, gender difference in depressive symptoms were reported, with female epilepsy patients being more affected [158]. In contrast, no gender difference in prevalence of depression was described, but males were more likely to be affected by psychosocial factors, while females were more influenced by epilepsy itself [159]. Experiments with rodents demonstrated that only male mice showed a longer immobility time in the FST, while both genders displayed anhedonia-like behavior [160]. Together these findings suggest that gender-specific care is needed to prevent or treat comorbidities in people with epilepsy.

Similar to the well-established sex differences in the prevalence of psychiatric disorders, the stress-induced neuroimmune priming in males and females, although comparable, is not identical [161]. Microglia isolated from females did not exhibit elevated cytokine responses in comparison to microglia isolated from males; however, microglia from both sexes showed reduced phagocytic activity [162]. Stress induces neuroinflammatory priming through multiple mechanisms, such as disruption of CD200R signaling involved in a persistent increase in HMGB1 [163,164] and its receptor RAGE [165], and autophagy inhibition in microglia promoting an exaggerated inflammatory response to a subsequent insult [166].

### 4.3. Neuroinflammation-Related Depression in Epilepsy

It is suggested that neuroinflammation-related depression might result from chronic stress exposure [70,167]. However, very few experimental studies have addressed the impact of stressors in epilepsy models and comorbid depression. Similar depressive-like behaviors were detected in rats subjected to LiCl-pilocarpine compared rats exposed to chronic unpredictable mild stress, a model of depression; however, gliosis was more robust in the hippocampus (a stress and seizure sensitive brain region) of SE rats [168]. These findings are consistent with the idea that chronic stress and depression in rodents results in worse epilepsy outcomes. Two papers published in 2015 provide more detailed information on stress-induced vulnerability to epilepsy and comorbid depression [24,26]. For instance, social defeat stress, followed by kainic acid-induced SE four weeks later, resulted in 50% of rats displaying a shorter latency to SE, accelerated epileptogenesis, and once epilepsy was induced, depressive-like behavior and cognitive deficits. Their results suggested a “two-hit scenario”, where the initial social defeat stressor (first hit) sensitizes a subset of rats, making them vulnerable to additional insults (second hit) [24]. Based on these findings, Maguire [26] proposed a model whereby exposure to previous stressors induces HPA axis hyperresponsiveness in a subset of animals; resulting in vulnerability when subjected to a “second hit,” at which time pathological consequences resulted in depressive-like behaviors and increased seizure susceptibility.

### 4.4. Neuroinflammation as a Target to Treat Epilepsy and Comorbid Depression

There is very limited evidence regarding the effectiveness of antidepressant agents on neuroinflammation and depressive symptoms associated with epilepsy (Table 1). Particularly in depressed patients, treatment with selective serotonin reuptake inhibitors (SSRIs) and serotonin-norepinephrine reuptake inhibitors (SNRIs), reduced pro-inflammatory cytokine, and increased anti-inflammatory cytokine production [169,170]. However, contrasting findings revealed either no effects of antidepressants on the inflammatory response associated with depressive symptoms [171], and others even exerted pro-inflammatory effects [172,173]. Future studies will need to investigate the anti-inflammatory effects of antidepressants in people with epilepsy and comorbid depression [135]. In rodents, the antidepressant fluoxetine was not effective at reducing spontaneous seizure occurrence nor improving depressive-like behaviors after pilocarpine-induced SE [148]. Interestingly, when fluoxetine was given in combination with an IL-1 receptor antagonist (IL-1ra), it significantly ameliorated depressive-like behaviors [174]. The efficacy observed in preclinical studies combining the administration of antidepressants with anti-inflammatory drugs suggest that combination therapy might be more effective to treat epilepsy and comorbid depression [175,176]. The antiepileptogenic effects of histone deacetylase inhibitors (HDACs) have been demonstrated in several rodent models of epilepsy [177]. Recently, HDACs, such as valproic acid (VPA) and sodium butyrate (NaB), proved to be effective at reducing the development of epilepsy [178,179] and improving comorbid depressive behavior in rodents [178]. Similarly, SAHA attenuated kainic acid-induced seizures, suppressed microglial activation in the hippocampus, and inhibited TLR4, MYD88, NF-κB, and IL-1β expression [180]. In primary microglia activated with lipopolysaccharide, VPA and NaB induced histone acetylation and enhanced prostaglandins release [181].

To date, several drugs targeting neuroinflammation have been used to reduce seizures, including COX-2 inhibitors, EP1 and EP2 receptors inhibitors, IL-1β inhibitors, and minocycline, among others (Table 1). As mentioned above, the inflammatory response in epilepsy is very complex and might differ among patients. These differences can possibly explain a significant burden of drug-resistance and emphasize the need for more targeted therapies. Moreover, the recent Fifteenth Eilat Conference on New Antiepileptic Drugs and Devices (EILAT XV) progress report summarizes key preclinical and phase 1 clinical data on new promising treatments to inhibit acute and chronic inflammation in epilepsy [182].

### 4.5. Potential Biomarkers in Epilepsy and Comorbid Depression

More investigation is needed regarding the diagnostic, predictive, and pharmacodynamic biomarkers of comorbidities in people with epilepsy [191,192]. If developed, biomarkers could provide useful tools to study stress responses, resilience, and vulnerability across species. An identified biomarker could potentially predict therapeutic efficacy in people most susceptible to seizures due to adverse life experiences [191]. Potential anti-inflammatory therapeutic targets for epilepsy were discussed recently [175]. These targets include COX-2 inhibitors and prostaglandin E2 receptor antagonists used to combat neuropathology after SE [127,187,193], as well as minocycline, the IL-1β synthesis inhibitor, and anti-TNFα antibodies. Unfortunately, accurate blood, prognostic, and neuroimaging biomarkers are still lacking.

### 4.6. Stress Primes the Brain to Depression

The mechanisms of stress are complex and not fully clarified. Exposure to stress induces peripheral immune cells to release danger signals, such as the alarmin HMGB1 [194]. In the first hit, HMGB1 sensitizes/primes microglia, the resident brain immune cells, to display a potentiated pro-inflammatory response to a subsequent insult [25,195]. Previous studies demonstrated that stress exacerbates microglial activation when followed by a subsequent insult, and worsens brain injury in stress-sensitive regions, such as the hippocampus, suggesting the presence of a “primed” pro-inflammatory microglial phenotype that affects pathological hallmarks of neurological disease [62,166,196,197,198,199]. This primed microglial phenotype is characterized by an increased immune machinery involved in an inflammatory response such as the NOD-like receptors; however, in the absence of a “second hit”, primed microglia do not release pro-inflammatory cytokines [163]. Furthermore, HMGB1 triggers primed microglia to upregulate the expression of the NLRP3 inflammasome, a complex required for pro-inflammatory cytokine production. Subsequent seizures (second hit) activate primed microglia and promote the inflammasome complex assemblage, and caspase-1 cleaves pro-IL-1β, resulting in secretion of mature IL-1β and leading to a potentiated inflammatory response and the development of spontaneous seizures (Figure 2).

The NLRP3 inflammasome is activated in peripheral blood immune cells obtained from depressed patients [200] and in the hippocampus from patients with epilepsy [201], and it has been implicated in the stress-induced inflammatory response associated with depressive-like behavior in rodents [202,203,204,205]. Together, these data suggest that stress-primed NLRP3 inflammasome activation could be a potential therapeutic target to treat depression in patients with elevated inflammation.

## 5. Conclusions and Future Directions

Screening and using early interventions for stress in epilepsy could improve diagnostics, treatments, and patients’ quality of life [10,24,206,207]. Stress management includes both pharmacological and non-pharmacological methods for enhancing stress coping mechanisms (e.g., mindfulness-based therapies, yoga, cognitive-behavioral therapies, etc.). In addition to healthy nutrition [208] and exercise [209], mindset approaches have been proposed to manage stress and depression in patients with epilepsy [210,211,212,213,214]. Notably, psychosocial interventions have been shown to improve the immune system function in humans [215]. Other studies also revealed a potential efficacy of anti-inflammatory drugs in the treatment of depression in both animal models of epilepsy and in people with epilepsy [21,216].

If stress indeed precipitates seizures, then therapy involving behavioral adjustment to reduce stress and transition a patient into a more relaxed state would help to reduce seizure frequency and in turn slow epilepsy progression and depression in patients with epilepsy. One confounding factor of this therapy is that stress is inherent to patients with epilepsy. Many patients suffering from epilepsy experience stress caused by the diagnosis and stigma of the disease, the risk of unpredictable seizures, and the lifestyle changes associated with preventing the occurrence of spontaneous seizures. In conclusion, although the relationship between neuroinflammation, stress, depression, and epilepsy is complex and still under investigation, it is important that healthcare professionals include stress management as a primary therapy along with seizure management, while treating epilepsy patients to improve epilepsy outcomes along with the psychiatric comorbidities.

## Figures and Tables

**Table 1:** Overview of studies targeting neuroinflammation to treat epilepsy and comorbid depression.

(**A**) Clinical studies			
**Drug**	**Mechanism of drug/target**	**Outcome**	**Ref**
Anakinra	IL-1ra	Reduced number of seizures	[183]
		Reduced peripheral blood monocytes cytokine production: IL-1β/IL-10 ratio	
Aspirin	COX-2 inhibitor	Reduced seizure frequency	[184]
		This study lacks of inflammation analysis	
Tocilizumab	IL-6 receptor	SE was terminated in most patients	[185]
		IL-6 levels were normalized	
		2 out of 7 patients experienced severe adverse events related to infection during therapy	
Minocycline	Microglial activation inhibitor	Reduced seizure frequency	[186]
		This study lacks of inflammation analysis	
(**B**) Preclinical studies			
Fluoxetine	Pilocarpine-induced SE	Failed to exert antiepileptogenic effects	[148]
		No improvement in depressive-like behaviors	
	SSRI	This study lacks of inflammation analysis	
Fluoxetine + IL-1ra	Pilocarpine-induced SE	Failed to exert antiepileptogenic effects	[174]
		Improved depressive-like behaviors	
	SSRI + IL-1ra	This study lacks of inflammation analysis	
VPA and NaB	Genetic model of absence epilepsy (WAG/Rij Rats)	Reduced absence seizures	[178,179]
		Improved depressive-like behaviors	
	Hippocampus kindling model	Inhibited development of kindling epileptogenesis	
	HDACs	These studies lack of inflammation analysis	
SAHA	Kainic acid-induced SE	Attenuated kainic acid-induced seizures	[180]
		Suppressed microglial activation in the hippocampus	
	HDAC	Inhibited TLR4, MYD88, NF-κB and IL-1β expression	
TG6-10-1	Organophosphorus-induced SE	Reduced hippocampal neuroinflammation and gliosis, mitigate neuronal injury or BBB breakdown	[127]
	EP2 antagonist		
TG8-260	Pilocarpine-induced SE	Reduced hippocampal neuroinflammation and gliosis but, in distinction to the earlier generation EP2 antagonist, did not mitigate neuronal injury or BBB breakdown	[187]
	EP2 antagonist		
Aspirin	Pilocarpine-induced SE	Reduced seizure frequency and duration	[188]
	COX-2 inhibitor	Reduced pro-inflammatory cytokine production: PGE2, IL-6 and TNF-α in the hippocampus	
Minocycline	Pilocarpine-induced SE	Inhibited microglial activation	[189]
		Reduced hippocampal neuroinflammation	
	Microglial activation inhibitor	Prevented neuronal loss	
		Reduced seizure frequency, duration, and severity	
GAO-3-02	Amygdala-kindled model	Reduced seizure severity	[182]
	Pilocarpine-induced SE	Reduced cognitive and memory deficits	
	Synaptamide	This study lacks of inflammation analysis	
miR-146a	Intra-amygdaloid injection of kainic acid	Reduced seizure progression and frequency	[190]
	IL-1R1/TLR4 activation	This study lacks of inflammation analysis	

## Supplementary Materials

The following are available online at https://www.mdpi.com/article/10.3390/ijms22084061/s1, Supplementary Table S1: Preclinical Studies Combining Acute Stress and Epilepsy, Supplementary Table S2: Clinical Studies Combining Acute Stress and Epilepsy.
