# Supplementary material for: Impact of Stress on Epilepsy: Focus on Neuroinflammation—A Mini Review
Source: Int J Mol Sci. 2021 Apr 14;22(8):4061. doi: 10.3390/ijms22084061 (PMC8071059; doi:10.3390/ijms22084061)
Supplement: Supplementary file 1 [file ijms-22-04061-s001.pdf]

International Journal of
Molecular Sciences

MDPI

Supplementary Table 1. Preclinical Studies Combining Acute Stress and Epilepsy

Stressor(s)	Species	Stress timing and duration	Epilepsy model	Stage of epilepsy progression being studied	Relevant outcomes	Reference
Conspecific alarm substance (CAS)	Zebrafish	Zebrafish in experimental group were exposed to 3.5 ml/L of CAS skin extract preparation for 5 mins. immediately before exposure to pentylenetetrazol	Pentylenetetrazol exposure	Epileptogenesis	CAS exposure increased seizure intensity, decreased latency to developing clonic-like seizure behaviors after PTZ exposure, and decreased the number of clonic-like seizure behaviors in CAS/PTZ zebrafish.	Canzian et al., 2021
Single and repetitive acute swim stress	CBA Mice	Experiment #1:<br>Mice exposed to 10 minutes of swim stress at 18-19 degrees Celsius 15 mins. before chemoconvulsant injection<br><br>Experiment #2:<br>Mice exposed to 10 minutes of swim stress	Picrotoxin, bicuculline, pentylenetetrazol, strychnine, kainic acid, or 4-aminopyridine injections	Epileptogenesis	Single acute swim stress increased dose threshold for convulsant signs and death for picrotoxin, pentylenetetrazol, strychnine, and 4-aminopyridine-induced seizures.<br><br>Single acute swim stress did not affect dose threshold for producing kainic-acid induced convulsions.	Pericic et al., 2001

		at 25 degrees Celsius (room temperature) 15 mins. before chemoconvulsant injection.<br><br>Experiment #3:<br>Mice exposed to repetitive swim stress two times a day for four consecutive days with one extra stress procedure on the fifth day; the last stress procedure was completed 25-15 mins. before chemoconvulsant injection.			Picrotoxin mice subject to room temperature swimming experienced a less robust stress effect than picrotoxin mice subjected to swimming in 18-19 degrees Celsius water.<br><br>Repetitive stress was less effective in reducing convulsive potency of picrotoxin than single acute stress.	
Acute restraint stress	NMRI mice	Plastic tube restraint stress lasting 30 mins., 1 hours, or 2 hours; restraint stress procedure completed 15 minutes before chemically induced seizure	Pentylenetetrazol infusion	Epileptogenesis	Mice exposed to 2 hours of restraint stress exhibited higher seizure thresholds than unstressed mice.	Homayoun et al., 2004

Acute foot shock stress	NMRI Mice	Prolonged intermittent foot shock (lasting 30 mins., 1-second-long shocks every 5 seconds) administered 1 min., 15 mins., 30 mins., and 60 mins. before chemically induced seizure	Pentylenetetrazol infusion	Epileptogenesis	Foot-shock stressed mice exhibited significantly higher seizure thresholds than non-stressed mice when given pentylenetetrazol infusion within 30 minutes after foot shock.	Shirzadian et al., 2018
Acute restraint stress	C7BL/6 Mice	Plastic tube restraint stress lasting 3 hours; One group of mice was subject to restraint stress prior to chemically induced seizure and the other group of mice subjected to restraint stress immediately after chemically induced seizure	Pilocarpine injection	Epileptogenesis	Mice stressed before pilocarpine injection exhibited a smaller reduction in paired pulse potentiation and long-term potentiation as compared to unstressed mice treated with pilocarpine.<br><br>Mice stressed after pilocarpine injection exhibited a larger reduction in paired pulse potentiation as compared to unstressed mice treated with pilocarpine.<br><br>Mice stressed after pilocarpine injection exhibited a rise in blood	Maggio et al., 2017 [

					corticosterone that lasted much longer than the rise seen in mice stressed before pilocarpine.<br><br>Pre-administration of a mineralocorticoid receptor agonist to unstressed pilocarpine-treated mice mimicked the effects of stress priming and decreased the reduction of LTP and paired pulse potentiation associated with pilocarpine.<br><br>Pre-administration of a mineralocorticoid antagonist to mice stressed before pilocarpine resulted in levels of reduction in long term potentiation and paired pulse potentiation that were similar to those of unstressed pilocarpine-treated mice.	
Repetitive acute swim stress	Adult mice	7 consecutive days of acute swim stress; each acute swim stress session lasted either 2	Pentylenetetrazol; Clonazepam pre-treatment for one group of mice	Epileptogenesis	Repetitive swim stress mice caused for less efficient binding of the benzodiazepine [3H]Ro15-1788 to its receptors in the	Weizman et al., 1989

		or 10 mins. and the last session was completed 30 minutes before pentylenetetrazol infusion			hippocampus, cerebral cortex, hypothalamus, midbrain, and striatum but not in the cerebellum, pons, and medulla.<br><br>Repetitive swim stressed mice exhibited reduced maximal binding capacity of benzodiazepine receptors when compared to unstressed mice.<br><br>The ED50 dose of clonazepam for protection against PTZ-induced seizures was higher for mice subjected to repeated swim stress.<br><br>None of the differences stated above were found using in vitro binding techniques.	
Acute repetitive social defeat stress	Wild-type Groningen (WTG) rats	4 days of repetitive social defeat stress ending 4 weeks prior to chemically induced seizure	Kainic acid	Epileptogenesis	One half of social defeat stressed mice exhibited a sustained reduction in BDNF levels.<br><br>Social defeat stressed mice with low BDNF levels exhibited	Becker et al., 2019

					reduction of seizure threshold and accelerated epileptogenesis than social defeat stressed mice with normal BDNF levels and unstressed mice.	
Acute repetitive social defeat stress	Wild-type Groningen (WTG) rats	4 days of repetitive social defeat stress ending 4 weeks prior to chemically induced seizure	Kainic acid	Epileptogenesis	Social defeat stressed mice with low BDNF levels exhibited reduction of seizure threshold and accelerated epileptogenesis, both of which were not observed in social defeat stressed mice with normal BDNF levels and unstressed mice.	Becker et al., 2015
Handling stress, immobilization stress, and conspecific threat stress	Hooded rats	Unspecified frequency and duration of stressors	Repetitive infusions of beta-endorphin and met-enkephalin	Chronic phase	Sporadic handling stress, immobilization stress, and conspecific threat stress exacerbated frequency and intensity of convulsive seizures.	Cain & Corcoran, 1984
Handling stress, conspecific threat stress	Hooded rats	Unspecified frequency and duration of stressors	Repetitive infusions of beta-endorphin and met-enkephalin	Chronic phase	Sporadic conspecific threat stress and handling stress exacerbated epileptiform spiking and convulsive signs for some, but not all epileptic rats.	Cain & Corcoran, 1985
Immobilization stress	Wistar Rats	Rats were subjected to immobilization by wire	Hippocampal kindling	Chronic phase	interictal discharges in the dorsal hippocampus were significantly	Takeshita et al., 1991

		netting for 1 hour starting 24 hours after electrical stimulation.			more frequent during immobilization stress. This effect disappeared during the 10 minutes after cessation of immobilization stress but returned during 10 to 60 minutes after stress cessation.<br><br>None of the hippocampal kindled rats exhibited generalized convulsions; however, some displayed behavioral arrest less than a minute after cessation of immobilization stress.	
Inter-male agonistic experience	TMD-S3 rats	Rats were subject to a resident/intruder social defeat paradigm during which an experienced "fighter" rat was introduced into the experimental rat's cage for 15 minutes.	Amygdala kindling	Chronic phase	Social defeat stressed rats had shorter and less severe motor seizures than unstressed animals.<br><br>The duration of postictal inhibition was also reduced by social defeat stress.	Beldhuis et al., 1992
Tail suspension stress	El mice and ddY control mice	One group of mice was suspended by the tail	El mice (Genetic model of idiopathic epilepsy)	Chronic phase	El mice had higher CRF peptide content in the paraventricular	Forcelli et al., 2007

Foot shock stress		for 2, 30 second periods, with a brief 15-minute break in between suspension sessions.<br><br>Another group of mice was subject to 2, 90-second-long foot-shock sessions, with 2-minutes in between the two sessions.			thalamic nucleus in response to tail suspension stress than ddY controls treated with the same stressor.<br><br>Plasma corticosterone levels for El mice were higher than ddY controls 60 min following handling and foot-shock stress.<br><br>El mice displayed greater EEG activity (1-4 Hz) in reaction to tail suspension stress than ddY controls.	
Acute restraint stress	Scn8a mutant mice	20-minute acute restraint stress using polypropylene restrainer immediately before chemically induced seizure	Scn8a mutation (Genetic model of absence epilepsy)<br><br>+<br><br>Picrotoxin or Kainic acid induced seizures	Chronic phase	Even before restraint stress, Scn8a mutants exhibited greatest seizure activity around time of peak corticosterone release (1700-1900 h).<br><br>Scn8a mutants showed more frequent spike wave discharges (SWDs) in response to chemoconvulsant injection	Sawyer et al., 2014

					immediately after restraint stress; however, wild type mice did not exhibit changes in SWD responses to chemoconvulsant after stress exposure.<br><br>Restraint stressed Scn8a mutants experienced more severe and longer lasting seizures in response to picrotoxin/kainic acid injection as compared to wild-type mice subject to the same stressor and chemoconvulsant.	
Repetitive foot shock stress	WAG/Rij rats, ACI rats, and Wistar rats	Experiment 1:<br>One day of foot shock stress with three electrical foot shocks delivered to rats with randomized inter-shock intervals lasting 1-10 seconds.<br><br>Experiment 2:	WAG/Rij rats (Genetic model of absence epilepsy)	Chronic phase	WAG/Rij and ACI rats had higher baseline levels of corticosterone as well as a quicker corticosterone response to foot shock stress.<br><br>WAG/Rij rats reached basal corticosterone levels more quickly than ACI and Wistar rats	Tolmacheva et al., 2012

		Three consecutive days of foot shock stress with one, three, or ten scrambled electrical shocks presented each day; each shock series (1,3,10) had an inter-shock interval of 1-10 seconds.			after exposure to foot shock stress.<br><br>WAG/Rij rats initially exhibited less spike wave discharges in response to foot shock stress, but eventually displayed exacerbated spike wave discharges preceding the third consecutive presentation of stressor.	

Suplemmentary Table 2. Clinical Studies Combining Acute Stress and Epilepsy

Stressor(s)	Patient population	Relevant methodology	Relevant findings	Reference
Patients surveyed for acute (lasting minutes-hours) and/or chronic (lasting days-months) stress precipitated seizures.	266 patients at tertiary epilepsy center	Patients were asked whether they experienced acute or chronic stress-precipitated seizures, and whether any stress reduction activities they've tried had been effective in limiting stress-precipitated seizures.	219/266 participants experienced stress-precipitated seizures. 68% of these 219 participants reported acute stress as a precipitant and 85% reported chronic stress.<br><br>57% of the 219 participants who experience stress-precipitated seizures tried stress reduction activities and 88% found them to be effective.	Privitera et al., 2014

Strong emotional stimuli by way of a stressful interview	39 participants total.<br><br>22 patients with psychomotor or psychosensory epilepsy.<br><br>8 patients who had previously experienced grand mal seizures.<br><br>9 non-epileptic controls.	EEG recordings were done for all participants before and during the stressful interview.<br><br>The stressful interview was led by the patient's physician and its contents were highly individualized. Each interview included interrogations, criticisms, accusations, laudatory comments, and apologies.<br><br>The authors noted that they were not completely successful in eliciting visibly emotional responses from all participants.	No significant change in the EEG was noted during the stressful interview in 5 patients.<br><br>20 patients showed abnormal EEG activity after the interview; for 9 of these 20 patients, the abnormal EEG activity included exaggerated spiking or recordings indicative of seizure activity.<br><br>Unprecedented pathological EEG activity was recorded in 11 patients.	Stevens, 1959
Personalized audio and video tapes of problematic social interactions	Patients with complex partial seizures (n = 5)	Patients were interviewed at length about their neurological	Video and audio replays of stress triggers were sufficient to induce seizures in all 5 patients.	Feldman et al., 1976

		background, the nature of their seizures, and whether or not they felt their seizures were precipitated by stressful stimuli. Additional background information on each patient was collected by means of contacting previous healthcare providers and family members, as well as by collecting current EEG data.<br><br>Patients are shown a series of personalized "stressor tapes" based on the stressful interpersonal relationships/situations they identified as triggers during the interview.		

High-intensity fitness program (CrossFit)	Case report: A patient with drug-resistant focal epilepsy (n = 1)	Patient attended 60 minute long high-intensity CrossFit classes three times a week.	After 6 months of engaging in weekly high-intensity exercise classes, the patient's seizure frequency dropped by 33%.<br><br>The patient's severity and duration of seizures did not change after starting high-intensity fitness program.	van der Kop et al., 2020 [33]
